# Status of Nuclear Medicine in Nepal: Current Infrastructure, Challenges, and Future Perspectives

**DOI:** 10.1055/s-0046-1823001

**Published:** 2026-05-22

**Authors:** Ajay Kumar Yadav, Om Prakash Yadav, Birendra Yadav, Amardeep Chaudhary, Ganga D. Adhikari, Bibekanand Aarya, Manish Tiwari

**Affiliations:** 1 Nuclear Medicine and Molecular Imaging Centre, Birat Medical College Teaching Hospital, Budhiganga-2, Morang, Nepal; 2 Department of Radio-Diagnosis, Imaging and Nuclear Medicine, BP Koirala Memorial Cancer Hospital, Bharatpur-7, Chitwan, Nepal; 3 Department of Nuclear Medicine, Chitwan Medical College, Bharatpur-10, Chitwan, Nepal; 4 Nepal Center of Nuclear Medicine, Lalitpur, Nepal

**Keywords:** Nepal, nuclear medicine, history, radioisotopes, SPECT, SPECT/CT, PET/CT, cyclotron

## Abstract

**Background:**

Nuclear medicine is an integral part of medical practice in Nepal. This article reports on the current infrastructure, human resources, training, and research related to nuclear medicine in Nepal.

**Methods:**

A questionnaire was sent to all nuclear medicine services in Nepal to collect data on the number and types of imaging devices, staffing, and procedures conducted in 2025. In addition, nuclear medicine–related publications were extracted from relevant research portals.

**Results:**

Nepal, a lower middle-income country of 30 million inhabitants, has nine nuclear medicine departments equipped with four single-photon emission computed tomography (SPECT), two SPECT/CT (computed tomography), and three positron emission tomography/CT (PET/CT) scanners. In 2025, nearly 200 scans per month were performed at each center with a gamma camera, whereas ∼100 PET/CT cases per month were performed at centers equipped with PET/CT scanners. A total of approximately 50 radionuclide therapy cases per month, mainly comprising I-131 therapies, were performed at centers with gamma cameras. Staff members include 9 MD nuclear medicine physicians, 3 MSc nuclear medicine technologists, 20 radiological technologists involved in nuclear medicine services, and a few staff nurses, although resources are unevenly distributed. Research output has increased substantially since 2020, often in collaboration with international institutions.

**Conclusion:**

Nepal has a relatively advanced nuclear medicine infrastructure supported by public investment, government regulation, international collaboration, and education. However, challenges remain in service expansion, workforce shortages, research development, and equitable access. If these challenges are addressed, Nepal could serve as a compelling model for improving nuclear medicine services in least-developed countries.

## Introduction

The history of nuclear medicine (NM) in Nepal (1988–November 2025) spans more than three decades. More than 37 years have passed since the first NM center was established at Bir Hospital, part of the National Academy of Medical Sciences (NAMS) in Kathmandu, the capital of Nepal. In 1988, NM was introduced at Bir Hospital with the installation of a gamma camera, the first of its kind in Nepal. 1

This marked the beginning of NM services in the country. In 1987, Dr. Gauri Shanker Pant, a medical physicist from the All India Institute of Medical Sciences, New Delhi, was sent to Nepal by the Indian government to establish and operate NM imaging services at Bir Hospital. He also contributed to the establishment of cobalt-60 facilities and trained Nepal's first medical physicist, Mr. Pradhuman Prasad Chaurasia. Approximately 20 years later, in 2009, Bir Hospital acquired a new single-photon emission computed tomography (SPECT) from Mediso (Hungary; AnyScan model). At that time, Dr. Ram Krishna Shrestha was a NM physician. The installation of the gamma camera was reportedly delayed due to procurement-related issues. Although the equipment arrived at the hospital on December 5, 2009, its installation was delayed, drawing the attention of the Legislature/Parliament. A subcommittee under the Women, Children, and Social Welfare Committee directed hospital management to bring the equipment into operation within 1 month. At that time, a NM facility at Metro Radiology Imaging Center (Naxal, Kathmandu; a private institution) had been established a few months earlier; however, it is no longer operational.

In 2008, Nepal became a member of the International Atomic Energy Agency (IAEA), marking a turning point in the recognition of medical physicists and fostering international collaboration in radiation medicine. The Radioactive Materials Use and Regulatory Act came into effect in July 2020, providing a stronger regulatory framework for the use of radioactive materials in Nepal. The aim of this cross-sectional study was to characterize the status of NM services across Nepal. The survey targeted all NM centers operating in the country as of October 2025, with all nine centers participating (100% response rate). The included centers represent the major types of equipment, including dual-energy X-ray absorptiometry, SPECT/computed tomography (CT), and positron emission tomography/CT (PET/CT) units, as well as radionuclide therapy services. The survey also assessed current infrastructure, human resources, training, and research pertaining to NM practice in Nepal. This article further assesses future perspectives of NM practice in Nepal.

## Methods

To obtain the necessary information, questionnaires were dispatched to hospitals via email. Departments that did not respond to our inquiry were contacted by telephone to obtain the necessary data. Hospitals with a NM department were requested to provide the following details: (1) basic information of personnel working in NM. (2) Number, models, and the age of each SPECT, SPECT/CT, PET, and PET/CT machine in current use. (3) Categories of quality control (QC) performed conventionally (daily, weekly, monthly, quarterly, and yearly) and their proportion. (4) Annual number of radioimmunoassay, frequency of main clinical applications for SPECT (or SPECT/CT) and PET/CT (or PET), and radioisotope therapies. (5) Radiological protection measures. The questionnaire on equipment and procedures asked for details of the imaging equipment available in each department and for the numbers of each type of diagnostic investigation or therapeutic treatment performed. We sought to provide beneficial information on the contemporary NM status and to analyze the prevailing trends of NM practice in Nepal. In addition, all NM-related publications were extracted from Google Scholar, ResearchGate, PubMed, Web of Science, and Elsevier to summarize all research articles related to NM practice in Nepal.

## Results

**Fig. 1:**
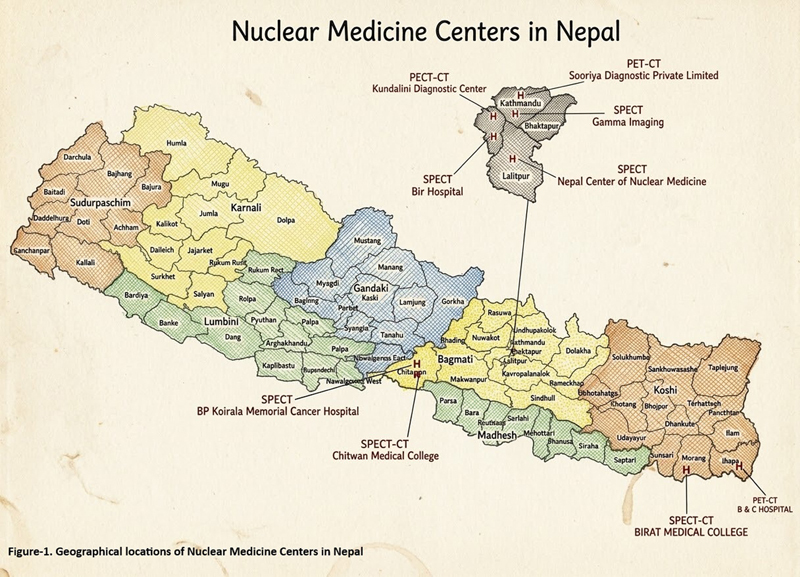
Geographical locations of nuclear medicine centers in Nepal.

A total of nine NM centers were identified across Nepal, of which seven (77.8%) were privately operated and two (22.2%) were government-run. These centers were located in major urban areas, including Kathmandu, Lalitpur, Biratnagar, Bharatpur, and Birtamode ( Fig. 1 ).

The total NM workforce comprised 45 professionals, including 8 MD NM physicians (17.8%), 2 PhD holders (4.4%), and 3 MSc graduates (6.7%). Additionally, six radiologists (13.3%) were involved in NM practice, along with a small number of Bachelor in Medical Imaging Technology graduates. Overall, there were 20 physicians/radiologists (44.4%) and 25 technologists (55.6%).

Regarding infrastructure, six centers (66.7%) were equipped with SPECT or SPECT/CT systems, while three centers (33.3%) had PET/CT scanners. Both government centers had nonfunctional equipment at the time of the study. All private centers (100%) had fully functional imaging systems.

All SPECT-equipped centers performed a wide range of diagnostic procedures. PET/CT centers performed whole-body 18F-FDG PET/CT scans exclusively. Therapeutic services were available in six centers (66.7%), all of which provided radioiodine therapy, while PET/CT-only centers did not offer therapy.

The mean number of SPECT scans per functional center was ∼187 ± 37 scans per month (range: 139–250). PET/CT centers performed a mean of 100 ± 0 scans per month. The total national monthly workload was ∼749 SPECT scans and 300 PET/CT scans.

Therapeutic procedures were performed in four centers, with a mean of 26 ± 10 cases per month per center (range: 12–35). The total number of radionuclide therapy procedures was ∼104 cases per month.

Two government centers (22.2%) were nonfunctional and did not contribute to clinical workload. Detailed characteristics of all centers, procedures, and workload distribution are summarized in Tables 1 to 3 .

**Table 1:** Detail descriptions of nuclear medicine centers in all over Nepal (services)

SN	Center and location	Staff (physician/tech)	Equipment and status	Services (diagnostic and therapeutic)
1	Nuclear Medicine & Molecular Imaging Centre, Birat Medical College, Biratnagar	1 NM physician, 2 NM technologists	SPECT/CT (GE NM/CT 850) and DEXA (Hologic Horizon); functional	All SPECT diagnostic tests; low- and high-dose radio-iodine therapy
2	Gamma Imaging and Research Center (GIRC), Kathmandu	3 NM physicians, 3 NM technologists	SPECT (Siemens Symbia); functional	All SPECT diagnostic tests; low- and high-dose radio-iodine therapy
3	Nepal Center of Nuclear Medicine, Lalitpur	2 NM physicians, 2 NM technologists	SPECT (GE NM 830); functional	All SPECT diagnostic tests; low- and high-dose radio-iodine therapy
4	Bir Hospital (NAMS), Kathmandu	2 NM physicians, 3 NM technologists	SPECT (Mediso AnyScan); not functional	Services currently not functional
5	Chitwan Medical College, Bharatpur	1 NM physician, 2 NM technologists	SPECT/CT (Siemens Symbia Intevo); functional	All SPECT diagnostic tests; low- and high-dose radio-iodine therapy
6	BP Koirala Memorial Cancer Hospital, Bharatpur	1 NM physician, 1 NM technologist	SPECT (Mediso AnyScan) and DEXA; not functional	Services currently not functional
7	Kundalini Diagnostic Center, Kathmandu	4 Radiologists, 4 NM technologists	PET/CT (Philips Ingenuity TF); functional	Whole Body 18F-FDG PET/CT; no therapeutics
8	Sooriya Diagnostic Private Limited, Kathmandu	4 Radiologists, 4 NM technologists	PET/CT (GE Discovery IQ); functional	Whole Body 18F-FDG PET/CT; no therapeutics
9	Purbanchal Cancer Hospital (B&C), Birtamode	2 Radiologists, 4 NM technologists	PET/CT (Siemens Biograph); functional	Whole Body 18F-FDG PET/CT; no therapeutics

Abbreviations: CT, computed tomography; PET, positron emission tomography; SPECT, single-photon emission computed tomography.

**Table 2:** The most frequently performed nuclear medicine procedures from Nepal

	Name of examination	Radionuclide used	Radiopharmacy used
**Diagnostic** **procedures**	Bone scan	^99m^ Tc	MDP
	Dynamic renal scan	^99m^ Tc	DTPA
	Static renal scan	^99m^ Tc	DMSA
	Thyroid scan	^99m^ Tc	
	Thyroid scan	^131^ I	
	Parathyroid scan	^99m^ Tc	SestaMIBI
	Cardiac scan	^99m^ Tc	SestaMIBI
	Oncology scan	^18^ F	FDG
**Therapeutic** **procedures**	Hyperthyroidism	^131^ I	Low-dose therapy
	Thyroid cancer	^131^ I	High-dose therapy

Abbreviations: DMSA, dimercaptosuccinic acid; DTPA, diethylenetriaminepentaacetic acid; FDG, fluorodeoxyglucose; MDP, methylene diphosphonate; MIBI, methoxy isobutyl isonitrile.

**Table 3:** Number of cases per months in 2025 (approximately) in different centers, Nepal

Name of centers	SPECT tests	PET tests	Therapeutic procedures
Nuclear Medicine & Molecular Imaging Centre, Birat Medical College, Biratnagar, Morang	**200**		**35**
Gamma Imaging and Research Center (GIRC), Maharajganj, Kathmandu	**200**		**32**
Nepal Center of Nuclear Medicine, Lalitpur	**210**		**25**
Department of Nuclear Medicine, Bir Hospital (NAMS), Kathmandu	**NIL**		**NIL**
Department of Nuclear Medicine, Chitwan Medical College, Bharatpur, Chitwan	**139**		**12**
Radiodiagnosis, Imaging & Nuclear Medicine, BPKMCH, Bharatpur, Chitwan	**NIL**		**NIL**
Kundalini Diagnostic Center, Kathmandu		**100**	
Sooriya Diagnostic Private Limited, Kathmandu		**100**	
Department of Nuclear Medicine (B&C), Birtamode, Jhapa		**100**	
Total	**749**	**300**	**104**

Abbreviations: PET, positron emission tomography; SPECT, single-photon emission computed tomography.

### Regulating Aspect of High-Dose Therapy

All nine NM departments prepared standard operating procedures (SOPs) to provide guidance on the management of patients/visitors and staff who may come into contact with patients receiving radionuclide therapy. The aim of the SOP is to deliver safe care to patients undergoing therapy, to ensure staff providing care for patients do so in a safe manner, to provide guidance enabling staff to provide appropriate information to patients and relatives, and to provide key contacts for further information. All the departments that provide targeted radionuclide therapy have well-facilitated and fully radiation-protected isolation wards with delayed tank toilets. 2 That special shielded isolation room has an attached private bathroom and all necessary facilities. Radiation-related warning signs are posted in that ward wherever necessary.

All potentially contaminated items (linens, food trays, and disposable personal items) are considered radioactive waste. They must be stored in designated containers within the room and managed by radiation safety personnel. The Radiation Safety Officer will measure the amount of radioactivity and complete the “radioactive waste for incineration” label and provide the nursing staff with three copies of a “Radioactive Clinical Waste Transfer Certificate.” Pregnant staff and children/minors are strictly prohibited from entering the ward. Radiation safety monitoring devices, which are common in all institutes, are summarized in Table 4 .

**Table 4:** Radiation safety monitoring devices that are common in all institutes

SN	Name	Measurement range	Calibration	Status
1	GM-Survey Meter	0.5 µSv/h to 40 mSv/h (0.05 mR/h to 4 R/h)	Half yearly	Functional
2	Pocket Dosimeter	0.5 µSv/h to 40 mSv/h	March 24, 2024	Functional
3	Dose Calibrator	N/A	February 2, 2020	Functional
4	TLD Badge	N/A	Every 3 months (BARC, India)	Functional

### Import of Radiopharmaceuticals in Nepal

The import of radiopharmaceuticals in Nepal is an area of the broader pharmaceutical import sector, which is highly regulated by the Department of Drug Administration under the Ministry of Health and Population. There is no such company that produces radioisotopes in Nepal. All of the NM centers import radioisotopes from other countries, i.e., India, Turkey, and France.

### Quality Assurance and Quality Control Procedures

Quality assurance is a crucial part of all aspects of NM practice. Almost all the centers that have SPECT are doing daily QC tests, i.e., background tests and image quality tests for both detectors. Background tests include energy peak (140 ± 10% KeV), full width at half-maximum ≤11.0%, uniformity cFoV ≤5.0%, uniformity uFoV ≤5.5%, total count ≤400,000 kcts for 1 minute, and count rate ≤45.00 kcts/s. Some of the centers also do weekly QC tests, i.e., uniformity, resolution/linearity tests, energy tests, and count density tests, but not all centers perform these QCs. Annual QC (COR test, whole-body quantitative SPECT/CT) is only performed by service engineers in Nepal. For SPECT radiopharmaceutical radiochemical purity (binding yield), most of the centers perform two-strip thin-layer chromatography. There is no automatic machine for QC of SPECT radiopharmaceuticals available in any centers.

Daily QC for PET/CT scanners in all three centers in Nepal is done with a fixed source, like a ^68^ Ge phantom or ^22^ Na point source, to verify system performance, ensuring image quality, quantitative accuracy, and proper functioning of the PET detectors and CT components. Annual QC for PET/CT is only performed by service engineers in Nepal.

### After-Sales Service and Maintenance of the Equipment

Ensuring the accuracy of biomedical engineering instruments in Nepali hospitals has become a significant concern, mainly due to the shortage of skilled biomedical engineering operations management teams. This issue is further complicated by the wide variety of imported biomedical equipment, which is not only diverse but often costly. Nepal's key challenge is to build an effective and capable system for managing the accuracy of biomedical devices so that their precision, durability, and reliability can be maintained.

The lack of organized biomedical engineering management teams has serious impacts on the health care sector, limiting its ability to provide high-quality and consistent patient services and ultimately affecting diagnostic accuracy. To address this issue, it is essential to develop a well-trained and efficient biomedical engineering operations management workforce in hospitals and health care facilities. This includes acquiring up-to-date skills for properly maintaining, testing, calibrating, and validating medical devices. Doing so will help extend the lifespan of these instruments while ensuring reliable and accurate diagnostics.

On the other hand, most of the companies, like General Electric (GE), Siemens, Philips, etc., develop their local service centers in different major cities of Nepal. The hospitals that installed the machines from this company have not faced too many problems with after-sales service and maintenance. There are two SPECT machines installed by Mediso Company in both government hospitals (Bir and BPKMC Hospital), which have faced too many problems in after-sales service and maintenance because there is no local service center from that company.

### After IAEA Membership in 2008

Nepal's membership in the IAEA in 2008 unlocked a range of significant benefits, primarily through the IAEA's Technical Cooperation Program. This support has focused on the peaceful application of nuclear science and technology across various sectors critical to the country's development. Support has been provided to improve equipment and facilities in NM and radiation oncology, including the procurement of advanced equipment like SPECT/CT systems for enhanced diagnostic accuracy. The IAEA has provided training, fellowships, and scientific visits to build local expertise in medical physics, NM, and radiation oncology.

Regarding radiation safety and security, a major pillar of cooperation has been the establishment of a robust national infrastructure for the safe and secure use of nuclear technology: the IAEA provides technical and legislative assistance, which has helped Nepal draft and enact national legislation related to the use and regulation of radioactive materials, such as the Radioactive Substances (Utilization and Regulation) Act, 2020. The IAEA helps in strengthening the overall radiation safety infrastructure, ensuring it aligns with international safety standards, including personal dosimetry, instrument calibration, and quality assurance of X-ray equipment.

### Publications Related to Nuclear Medicine

There is no journal exclusively dedicated to “NM” in Nepal. The research articles related to the NM field in Nepal are published in a few Nepalese medical and radiology journals that cover a broader scope and international journals. Getting a precise, up-to-date, total number of published research articles specifically on NM in Nepal is challenging, as there is no single global database that provides this exact statistic.

However, based on available information, we can make some key observations about the volume and nature of research coming out of Nepal in this field. It is growing but limited in number. The overall volume of medical and health-related research in Nepal is generally considered minimal when compared with developed nations. A review on occupational radiation safety in Nepal published in 2022, for example, could only find 15 original research articles on that topic published between 2007 and 2020. This suggests that the subset of research focused specifically on NM procedures and clinical outcomes is likely also modest in number. Focus on Radiation Safety and Infrastructure: A good portion of the documented published work related to nuclear technology in the Nepalese medical sector often focuses on foundational issues like radiation protection status, radiation safety, and the need for infrastructure and regulatory improvements, rather than large-scale clinical trials.

Some relevant articles are published in international journals and Nepalese Journal of Radiology (NJR), which specifically aims to share knowledge among radiologists, NM physicians, and physicists in Nepal.

In summary, while an exact figure is not publicly available, the total number of peer-reviewed research articles specifically on clinical NM in Nepal is likely in the dozens to low hundreds, with an increasing trend as NM services, like PET-CT, are expanded in the country.

## Future Projects of Nuclear Medicine in Nepal

Lots of hospitals are planning to have NM facilities, and also some hospitals that already have SPECT or SPECT-CT are planning to extend their NM facilities by adding PET-CT or PET-CT with a medical cyclotron. The Birat Medical College Teaching Hospital has already announced that it will start the PET/CT service very soon. In the same way, lots of institutes are planning to add PET-CT service, i.e., Bhaktapur Cancer Hospital, BP Koirala Memorial Cancer Hospital (BPKMCH), Tribhuvan University Teaching Hospital, Chitwan Medical College, and BP Koirala Institute of Health Sciences.

In 2013, Nepal's first honorable president, Ram Baran Yadav, had to travel to Japan for a PET scan after a black spot was detected in his intestine, as the service was unavailable in Nepal at the time. Upon his return, he shared his experience and asked the government to allocate funds to purchase the necessary equipment for Nepal. The government did allocate funds (up to Rs 400 million in one fiscal year) to buy PET/CT with a cyclotron facility, but the tender process for the machine at the BPKMCH faced delays, and the budget froze at the time. Now, there are three private PET-CT centers available in Nepal, but the country still lacks the first-ever medical cyclotron to supply radiopharmaceuticals for PET-CT scans. This facility will allow for the local production of medical isotopes for PET scans, which is crucial for advanced cancer diagnosis and treatment, thereby reducing the reliance on imported materials and eliminating the need for patients to travel abroad for essential scans.

## Discussion

This study provides a comprehensive cross-sectional analysis of the current state of NM services, infrastructure, human resources, and workload across Nepal in 2025. The findings highlight significant growth in the sector, primarily driven by private investment, yet they also reveal critical disparities and developmental gaps that require immediate policy attention. Since its introduction in 1988 at the NAMS, Bir Hospital, the field has expanded to nine operational centers, a clear indicator of growing recognition and demand for advanced diagnostic and therapeutic services. 1 Nepal's milestone achievement of joining the IAEA in 2008 and the subsequent materialization of the Radioactive Materials Uses and Regulatory Act in 2020 provide a necessary regulatory framework for this expansion.

A key finding is the distinct disparity in functionality and service provision between public and private NM centers. Of the nine centers, seven are private, and they are exclusively responsible for the current clinical workload. The data demonstrate that both government-run facilities, the Department of Nuclear Medicine at Bir Hospital (NAMS) and the Department of Radiodiagnosis, Imaging, and Nuclear Medicine at BPKMCH, possess nonfunctional SPECT equipment. This severe lack of functional infrastructure in the public sector forces patients into the private health care system, potentially creating significant access and affordability barriers for the wider population, especially those requiring complex procedures like radio-iodine therapy.

Furthermore, the geographical distribution reveals a concentration of services in major urban centers (Kathmandu, Lalitpur, Biratnagar, Bharatpur, and Birtamode). While the establishment of a PET/CT center in Birtamode, Jhapa, indicates a positive move toward decentralization in the Eastern region, a majority of the population still lacks reasonable access to these advanced diagnostic facilities, necessitating travel and increasing the overall burden of care.

The total NM workforce of 45 professionals highlights a relatively small, yet highly specialized, professional pool serving the entire country. The composition includes NM physicians and NM technologists demonstrating a foundational level of expertise. However, a significant observation from Table 1 is the reliance on radiologists (a total of six) to fill the NM physician role in certain centers, particularly those primarily offering PET/CT services. While collaboration between NM and radiology is essential, the long-term sustainability and quality assurance of specialized NM interpretation may require a greater focus on training and retaining dedicated NM physicians. The finding of 20 NM physicians/radiologists and 25 technologists suggests a reasonable clinical ratio, but the small total number underscores the vulnerability of the service should any professional leave the country or the field.

The analysis of caseloads provides valuable insight into the clinical demand. The total estimated monthly workload includes approximately 750 SPECT diagnostic tests, 300 PET diagnostic tests, and 100 therapeutic procedures per month.

A critical limitation for sustained growth is the complete dependence on imported radioisotopes from countries like India, Turkey, and France. This reliance exposes the entire sector to risks of supply chain disruption, high costs, and short half-life challenges (especially for those used in PET scans). The announcement in 2025 to establish Nepal's first cyclotron facility in Nepal will be a hope to strengthen the NM field; therefore, it is the single most important future development. Successful implementation of this facility will be transformative, enabling local production of key isotopes, significantly reducing costs and logistical complexity, and ensuring the long-term viability of advanced NM services like PET/CT.

The Nuclear Medicine Global Initiative, established in 2012 by 13 international organizations, is dedicated to promoting human health by advancing the practice of NM and molecular imaging. 3 The initiative's work has brought to light an array of international issues concerning radiopharmaceuticals, including regulatory barriers, cost, availability, and supply chain difficulties. 3 A particularly unexpected finding was the scarcity of standard diagnostic radiopharmaceuticals in numerous low- and middle-income nations. Given the global expansion of NM, addressing these access and availability challenges is a crucial strategy for maximizing patient benefit from these essential imaging and therapeutic methods. 3

The limited volume of research articles on NM, as indicated by its publication only in broader medical and international journals, points to a need for greater academic output. The focus of existing literature on foundational issues like radiation safety and infrastructure is understandable given the nascent stage of the field, but future growth must be accompanied by increased clinical research to optimize local protocols, assess treatment outcomes, and contribute to the global evidence base. The establishment of NJR is a positive step toward providing a dedicated platform for local research dissemination.

The current state of NM in Nepal is one of vigorous, private-sector-led growth juxtaposed with crippled public-sector infrastructure and a vital dependence on international supply chains. While private centers have successfully brought advanced PET/CT and SPECT/CT technology and services to the country, the nonfunctional status of equipment in government hospitals must be urgently addressed to ensure equitable access. Future development must prioritize the rapid implementation of the planned cyclotron facility to ensure radiopharmaceutical self-sufficiency.

Finally, when we see the global perspective and compare it with the status of NM in other countries, Nepal still needs revolutionary growth in the NM field like other countries. 4 5 Nepal is still at its beginning stage of NM field development.

## Conclusion

The NM sector in Nepal, 37 years after its inception, is currently in a state of rapid expansion and transition, primarily driven by private health care investment. The country now hosts nine centers, with seven private facilities providing the entire clinical workload, including advanced PET/CT and SPECT/CT diagnostics, and essential therapeutic procedures. This growth signifies a major positive development in the medical field across the nation. On the other hand, failure in the government sector and geographic disparity, i.e., services in major urban hubs, create significant access hurdles for the population in remote and rural areas.

The proposed cyclotron facility in Nepal is a necessary and transformative step that, if successfully implemented, will address the core issue of radiopharmaceutical dependency. Moving forward, national policy must prioritize restoring functionality to government centers and investing in localized training programs for NM physicians and technologists to ensure the sustainability, quality, and equitable distribution of these life-saving services across Nepal.
